# Latitude Determination and Error Analysis for Stationary SINS in Unknow-Position Condition

**DOI:** 10.3390/s20092558

**Published:** 2020-04-30

**Authors:** Suier Wang, Gongliu Yang, Wei Chen, Lifen Wang

**Affiliations:** 1 School of Instrumentation Science and Opto-Electronic Engineering, Beihang University, Beijing 100191, China; xhwangsuier@buaa.edu.cn (S.W.); yanggongliu@buaa.edu.cn (G.Y.); 2 Department of Aerospace Science and Technology, Space Engineering University, Beijing 101416, China; wanglifen_2009@139.com

**Keywords:** latitude determination, strapdown inertial navigation systems, gyros bias, coarse alignment

## Abstract

The initial geographic latitude information is the key to the self-alignment of the strapdown inertial navigation system (SINS), but how to determine the latitude when the latitude cannot be obtained directly or in a short time? The latitude determination (LD) methods are introduced, including magnitude method, geometric method, and analytical methods 1 and 2, to solve this situation only by the output of the SINS itself. Simulation and experimental test results validate the efficiency of these LD methods. In order to improve the accuracy of the LD, the error of the LD method is derived through comparative analysis. Based on the relationship between LD error and inertial measurement unit (IMU) bias. Partial bias estimation method is introduced and executed during latitude determination. After compensating the estimated IMU bias, the accuracy of the LD will be further improved. Latitude errors are also affected by the latitude where SINS is located. Comprehensive simulation and experimental tests verify the effectiveness of the method. The IMU determined latitude can not only be used to achieve the self-alignment of the SINS, but also to correct the navigation latitude of the long-term SINS, thereby improving the autonomy and positioning accuracy of the navigation system.

## 1. Introduction

A strapdown inertial navigation system (SINS) can provide entirely autonomous altitude, velocity and positioning navigation solutions [1]. The inertial measurement unit (IMU) consists of three-axis orthogonal gyroscope and accelerometer components, used to measure the angular velocity and specific force of vehicles, missiles, aircraft and ship [2]. Compared with other navigation systems such as global navigation satellite system (GNSS) and astronomical navigation system, the inertial navigation system (INS) provides a navigation solution that is all-weather, high-frequency, continuous and not affected by the external environment.

Generally, the start-up of an INS is also known as the typical initial alignment [3], which consists of two phases: coarse alignment (CA) and fine alignment (FA), respectively. The accurate location information especially geographic latitude is a crucial prerequisite for the existing SINS initial alignment methods. Without geographic latitude, even an SINS can not work and provide navigation solutions, which limits some applications of SINS [4]. At present, the positioning information is obtained by GNSS for self-alignment of INS. However, there are few studies on how to achieve alignment for applications that cannot receive GNSS satellite signals, such as tunnels, underground, forests, underwater, canyons, and other places that interfere with satellite information, and other application environments where it is difficult to obtain accurate geographic latitudes.

Whereas, the commonly used navigation coordinate frame (*n*-frame) is established based on the SINS location (latitude, longitude, and altitude), and its axes $x_{n}$, $y_{n}$, and $z_{n}$ point to the east, north and up (ENU), known as the local geographic coordinate frame. In addition, the amplitude of gravity acceleration changes with the change of geographic latitude. The gravity acceleration of P point on surface of the Earth can be obtained by $g_{p}=9.78049\left(1+0.0052884\sin^{2}L-0.0000059\sin^{2}2L\right)$, where *L* is the geographic latitude [5]. Because the gravity acceleration is a major function of latitude that latitude information is very important for calculating the magnitude of local gravity acceleration. Moreover, the geographic latitude can be used to decompose the Earth rate vector into *n*-frame. In *n*-frame, the projection of the Earth rate vector in three axes is [ 0, ΩcosL, ΩsinL${]}^{T}$ (Ω is the amplitude of the Earth rate vector) respectively, while the acceleration vector of gravity is only in the z-axis direction, and the amplitude is equal to the local gravity [6]. Therefore, the initial geographic latitude is significant and indispensable for INS, so how to determine the geographic latitude in these applications mentioned above is a core task. When there is only an INS, can we only use the static measurement information of the IMU to obtain the geographic latitude?

Inspired by the analytical CA method (also known as the three-axis attitude determination (TRIAD)) [7,8,9], the gyros triads measure the Earth’s rate vector while the accelerometers triads measure the local gravity acceleration vector in body frame (*b*-frame) when the SINS is static [10,11]. Therefore, the geographic latitude is determined by using the static constraints of SINS and the measurement of IMU, and the four geographic latitude determination (LD) methods are elaborated in the paper. In [12], the derivation process of geometric LD is given. Yan’s work is mainly to estimate the latitude for initial alignment, the error analysis of LD is not his interest. Aiming at the alignment problem of SINS without latitude, Refs. [13,14] provide a method based on the quaternion calculation of vector of earth rotational angular velocity to achieve initial alignment for both stationary and swaying base. Even if the initial alignment without latitude can be achieved, without the latitude information, the system cannot continue the navigation task. Refs. [15,16] proposed a method for latitude estimation and initial alignment on swaying base. These works did not analyze the latitude estimation error and the latitude determination algorithm is not comprehensive.

This paper mainly expends four LD methods for the stationary SINS in unknown-position condition. By the error analysis of LD methods, it is concluded that the estimated latitude error is mainly affected by the biases of the accelerometers and gyros, or some certain direction gyros and accelerometers bias in navigation frame. The analytical CA and gyro bias estimation method by two position alignment was proposed in [17]. Using the two different attitudes of INS, the three-axis gyros bias can be calculated. The estimation and compensation of the gyros biases can effectively reduce the aligned heading misalignment angle and improve the navigation accuracy. Silva analyzes normality and orthogonality error of the analytical CA DCM from *b*-frame to *n*-frame $C_{b}^{n}$ then gives the bias of northward and upward gyros and upward accelerometer bias estimation [18]. After the LD process and initial alignment can be performed simultaneously, the estimated latitude can be used synchronously for the INS to quickly start without the initial position. Based on the LD error analysis, the CA bias estimation method (CA-CBE) is introduced. After compensating the bias, the accuracy of latitude estimation is improved obviously. Furthermore, the relationship between latitude error and latitude of four methods is analyzed, so that the best LD method can be selected in different locations. In addition, the determined latitude can correct the position error of the inertial navigation system that works for a long time.

This paper is organized as follows. Section 2 gives four LD methods, whilst the comprehensive error analysis of each LD method is proposed in Section 3. The IMU partial bias estimation algorithm is drawn into Section 4. Section 5 and Section 6 present simulation and experiment results. Finally, the conclusion is devoted in Section 7.

## 2. Latitude Determination Methods

### 2.1. Analytic Coarse Alignment Algorithm

The purpose of CA of the SINS is to calculate the attitude matrix, which relates the body frame and the navigation frame. The analytic CA performed based on computing a set of three non-collinear vectors ${\left[f^{b},\;\omega_{ib}^{b},\;{\left(f\times \omega_{ib}\right)}^{b}\right]}^{T}$ in the body frame and a set of three non-collinear vectors ${\left[g^{n},\;\omega_{ie}^{n},\;{\left(g\times \omega_{ie}\right)}^{n}\right]}^{T}$ in the navigation frame [3,8]. The body frame is denoted by *b* with its axes $x_{b}$, $y_{b}$ and $z_{b}$ pointing to the right, forward, and upward directions (RFU), respectively. The attitude matrix can be expressed as

(1) $$C_{b}^{n}={\left[\begin{array}{c} g_{}^{n^{T}}\\ \omega_{ie}^{n^{T}}\\ {\left(g\times \omega_{ie}\right)}^{n^{T}} \end{array}\right]}^{-1}\left[\begin{array}{c} f_{}^{b^{T}}\\ \omega_{ie}^{b^{T}}\\ {\left(f\times \omega_{ie}\right)}^{b^{T}} \end{array}\right]$$

It is assumed that the INS is located at point *P* on the surface of the Earth. Where $g^{n}={\left[0,\;0,\;-g_{p}\right]}^{T}$, $f^{b}={\left[f_{x},\;f_{y},\;f_{z}\right]}^{T}$, $\omega_{ie}^{n}={\left[0,\;\Omega \cos L,\;\Omega \sin L\right]}^{T}$, $\omega_{ie}^{b}\approx \omega_{ib}^{b}={\left[\omega_{x},\;\omega_{y},\;\omega_{z}\right]}^{T}$, Ω is the magnitude of the earth rate, *L* is the latitude of *P* and $g_{p}$ is the gravity acceleration of *P* somewhere on the earth, $C_{b}^{n}$ can also be expressed as:

(2) $$\begin{array}{c} C_{b}^{n}=\left[\begin{array}{ccc} \frac{\omega_{z}f_{y}-\omega_{y}f_{z}}{g_{p}\Omega \cos L} & \frac{\omega_{x}f_{z}-\omega_{z}f_{x}}{g_{p}\Omega \cos L} & \frac{\omega_{y}f_{x}-\omega_{x}f_{y}}{g_{p}\Omega \cos L}\\ \frac{f_{x}\tan L}{g_{p}}+\frac{\omega_{x}}{\Omega \cos L} & \frac{f_{y}\tan L}{g_{p}}+\frac{\omega_{y}}{\Omega \cos L} & \frac{f_{z}\tan L}{g_{p}}+\frac{\omega_{z}}{\Omega \cos L}\\ -\frac{f_{x}}{g_{p}} & -\frac{f_{y}}{g_{p}} & -\frac{f_{z}}{g_{p}} \end{array}\right] \end{array}$$

The DCM of the attitude matrix $C_{b}^{n}$ is given.

(3) $$\begin{array}{c} C_{b}^{n}\;=\;\left[\begin{array}{ccc} c\gamma c\psi \;+\;s\gamma s\theta s\psi & c\theta s\psi & s\gamma c\psi \;-\;c\gamma s\theta s\psi \\ -c\gamma s\psi \;+\;s\gamma s\theta c\psi & c\theta c\psi & -s\gamma s\psi \;-\;c\gamma s\theta c\psi \\ -s\gamma c\theta & s\theta & c\gamma c\theta \end{array}\right] \end{array}$$

where cx represents cosx and sx represents sinx, where x=θ,γ,ψ. Combining Equation (2) with Equation (3), pitch θ, roll γ, and azimuth ψ are calculated by the following equation

(4) $$\begin{array}{c} \left\{\begin{array}{c} \gamma =-\arctan\frac{C_{31}}{C_{33}}=\arctan\frac{f_{y}}{f_{z}}\\ \theta =\arcsin C_{32}=-\arcsin\frac{f_{y}}{g_{p}}\\ \psi =-\arctan\frac{C_{21}}{C_{22}}=\arctan\frac{\omega_{y}f_{x}-\omega_{x}f_{y}}{g_{p}\omega_{y}-f_{y}\Omega \sin L} \end{array}\right. \end{array}$$

The analytic CA process is to calculate the coarse attitude for the FA process, which is an indispensable stage for the initial alignment of the static INS. It can be seen from the analytic CA, the geographic latitude is indispensable, because the representation of $\omega_{ie}^{n}$ in the *n* frame has a direct relationship with the geographic latitude; in addition, different latitudes and different heights will affect the local gravitational acceleration $g_{p}$ [5], and the relationship is shown in the following equation.

(5) $$\begin{array}{c} \begin{array}{c} g_{p}=9.78049\left(1+0.0052884\sin^{2}L-0.0000059\sin^{2}2L+\right)-0.000003086h \end{array} \end{array}$$

where *h* is the height of SINS. The main research purpose of this paper is to determine the latitude, and the height can be obtained by barometer.

### 2.2. Magnitude Latitude Determination Method

The measurement of IMU can be expressed into *n* frame by $C_{b}^{n}$ when the INS is perfectly stationary, as well as the error and noise of inertial devices are not considered [5,6].

(6) $$g^{n}=C_{b}^{n}f^{b}$$

(7) $$\omega_{ie}^{n}=C_{b}^{n}\omega_{ib}^{b}$$

The inner product of the two vectors can be obtained as follows.

(8) $$\left\{\begin{array}{c} {\left(g^{n}\right)}^{T}\omega_{ie}^{n}=\Omega g_{p}\sin L\\ {\left(f^{b}\right)}^{T}\omega_{ib}^{b}=f_{x}\omega_{x}+f_{y}\omega_{y}+f_{z}\omega_{z} \end{array}\right.$$

The latitude *L* can be can be expressed by the IMU outputs as follows.

(9) $$L=\arcsin\left(\frac{f_{x}\omega_{x}+f_{y}\omega_{y}+f_{z}\omega_{z}}{g_{p}\Omega }\right)$$

Due to the unknown position of the IMU, it is considered that the gravity acceleration at point *P* cannot be calculated according to the gravity model as Equation (5). Therefore, the global average gravity acceleration $\bar{g}=9.80665$ is used here instead of the gravity acceleration $g_{p}$ at point *P*. Then latitude *L* can be can be expressed as follows.

(10) $$L=\arcsin\left(\frac{f_{x}\omega_{x}+f_{y}\omega_{y}+f_{z}\omega_{z}}{\bar{g}\Omega }\right)$$

### 2.3. Geometric Latitude Determination Method

**Figure 1:**
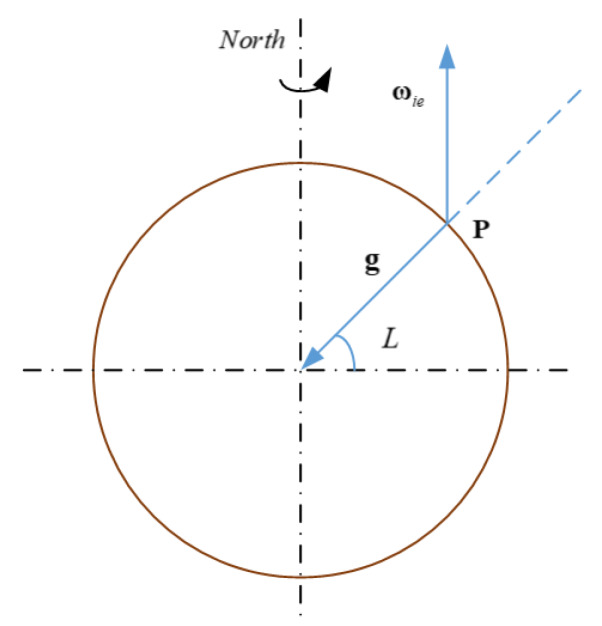
Representation of the latitude, local gravity acceleration vector and Earth rotation angular velocity vector at point P.

Under the assumption of the spherical model of the Earth, as shown in Figure 1, the geographic latitude is defined as the complementary angle between the Earth’s rate vector and the gravity vector. The geometric method determines the latitude based on this definition.

According to [12], the latitude *L* at *P* point on the Earth’s surface can be expressed by the following equation.

(11) $$L=\frac{\pi }{2}-\arccos\left(\frac{-g^{T}\omega_{ie}}{\left|g\right|\left|\omega_{ie}\right|}\right)$$

Simultaneously performing sines operators on both sides of the above equation

(12) $$\sin L=\frac{-g^{T}\omega_{ie}}{\left|g\right|\left|\omega_{ie}\right|}$$

Suppose there is a stationary SINS without considering its error and noise, that is $f^{b}=-g^{b}$ and $\omega_{ie}^{b}=\omega_{ie}^{b}=\omega_{ib}^{b}$, then the latitude equation becomes

(13) $$L=\arcsin\left(\frac{{\left(f^{b}\right)}^{T}\omega_{ib}^{b}}{\left|f^{b}\right|\left|\omega_{ib}^{b}\right|}\right)$$

where |x| denotes the length of the vector x, that is equal to the 2-norm of the vector.

### 2.4. Analytic Latitude Determination Method

The gyros triad measures the vector of the Earth’s rate, while the accelerometers triad measures the vector of local gravity acceleration when the SINS is static, that is

(14) $$f^{b}=C_{n}^{b}g^{n}$$

where $C_{n}^{b}$ is the DCM from *n* frame to *b* frame, and $C_{n}^{b}={\left(C_{b}^{n}\right)}^{T}$. Expanding Equation (14) can obtain the following equation.

(15) $$\left\{\begin{array}{c} f_{x}=-s\gamma c\theta \cdot g_{p}\\ f_{y}=s\theta \cdot g_{p}\\ f_{z}=c\gamma c\theta \cdot g_{p} \end{array}\right.$$

where $g_{p}\approx \sqrt{f_{x}^{2}+f_{y}^{2}+f_{z}^{2}}$, and expanding Equation (7) can obtain the following equation.

(16a) $$\begin{array}{c} \left(c\gamma c\psi +s\gamma s\psi s\theta \right)\omega_{x}+\left(s\psi c\theta \right))\omega_{y}+\left(s\gamma c\psi -c\gamma s\psi s\theta \right)\omega_{z}=0 \end{array}$$

(16b) $$\begin{array}{c} \left(s\gamma c\psi s\theta -c\gamma c\psi \right)\omega_{x}+\left(c\psi c\theta \right)\omega_{y}-\left(s\gamma s\psi +c\gamma s\psi s\theta \right)\omega_{z}=\Omega \cos L \end{array}$$

(16c) $$\left(-s\gamma c\theta \right)\omega_{x}+\left(s\theta \right)\omega_{y}+\left(c\gamma c\theta \right)\omega_{z}=\Omega \sin L$$

#### 2.4.1. Analytic 1 Method

The initial pitch θ and roll γ can be obtained from Equation (16).

(17) $$\left\{\begin{array}{c} \theta =\arcsin\left(f_{y},\sqrt{f_{x}^{2}+f_{y}^{2}+f_{z}^{2}}\right)\\ \gamma =\arctan\left(-f_{x},f_{z}\right) \end{array}\right.$$

Bringing θ and γ into Equation (16c), then latitude can be calculated as follows, that is called the analytical 1 method.

(18) $$L=\arcsin\frac{\left(-s\gamma c\theta \right)\omega_{x}\;+\;\left(s\theta \right)\omega_{y}+\left(c\gamma c\theta \right)\omega_{z}}{\Omega }$$

#### 2.4.2. Analytic 2 Method

The azimuth ψ can be computed combine the Equation (16a) with Equation (17) as follows.

(19) $$\psi =\arctan\left(-\frac{c\gamma \omega_{x}+s\gamma \omega_{z}}{s\gamma s\theta \omega_{x}+c\theta \omega_{y}-c\gamma s\theta \omega_{z}}\right)$$

The latitude can be calculated by dividing both sides of Equation (16c) by Equation (16b).

(20) $$\begin{array}{c} \begin{array}{c} L=\arctan(-s\gamma c\theta \omega_{x}+s\theta \omega_{y}+c\gamma c\theta \omega_{z},\left(s\gamma c\psi s\theta -c\gamma c\psi \right)\omega_{x}+c\psi c\theta \omega_{y}-\left(s\gamma s\psi +c\gamma c\psi c\theta \right)\omega_{z}) \end{array} \end{array}$$

where θ and γ can be obtained from Equation (17).

## 3. Latitude Determination Error Analysis

The previous section derived the LD formula based on the IMU error-free assumption, but for the actual INS, the inertial sensor error usually includes bias, random walk, scale factor error and bias drift [1,2]. However, it takes only a few minutes to complete the LD, and calculating the average value can effectively reduce the influence of noise, therefore the in-run biases of the IMU are considered as the main error source for LD. In addition, when the INS is static, the IMU’s scale factor error is negligible.

The in-run biases of gyros and accelerometers are $\delta \omega^{b}$ and $\delta f^{b}$, respectively, that is

(21) $$\left\{\begin{array}{c} {\hat{\omega }}_{b}=\omega_{b}+\delta \omega_{b}\\ {\hat{f}}_{b}=f_{b}+\delta f_{b} \end{array}\right.$$

To unify the error analysis of the latitude estimation, the equivalent biases in the *n* frame are analyzed as the error source, since the *b* frame can be arbitrarily oriented with respect to the *n* frame. For example, ${f_{b}}^{T}\delta \omega_{b}={\left(C_{n}^{b}f^{n}\right)}^{T}C_{n}^{b}\delta \omega^{n}={\left(f^{n}\right)}^{T}\delta \omega^{n}$, and $\sin L={f_{b}}^{T}\omega_{b}/g_{p}\Omega$ for ideal case. The expressions become

(22) $$\left\{\begin{array}{c} {f_{b}}^{T}\delta \omega_{b}=g_{p}\delta \omega_{U}\\ {\omega_{b}}^{T}\delta f_{b}=\Omega \cos Lg_{p}\delta f_{N}+\Omega \sin Lg_{p}\delta f_{U}\\ {\omega_{b}}^{T}\delta \omega_{b}=\Omega \cos L\delta \omega_{N}+\Omega \sin L\delta \omega_{U}\\ {f_{b}}^{T}\delta f_{b}=g_{p}\delta f_{U} \end{array}\right.$$

where the subscripts *N*, *U* represent the amount of error in the ENU direction.

### 3.1. Magnitude Latitude Determination Method

The Equation (10) of magnitude LD method can be rewritten as follows

(23) $$\sin L=\frac{{f_{b}}^{T}\omega_{b}}{\bar{g}\Omega }$$

Substituting Equation (21) into Equation (23) yields

(24) $$\sin\left(L+\delta L\right)=\frac{1}{\bar{g}\Omega }{\left(f_{b}+\delta f_{b}\right)}^{T}\left(\omega_{b}+\delta \omega_{b}\right)$$

Assuming that the latitude error is a small angle error, where δL is the latitude error, expand the above equation and ignore the second-order error term. The above equation combined with Equation (22) can be simplified to obtain the following equation.

(25) $$\delta L\approx \left(k_{g}-1\right)\tan L+k_{g}\left(\frac{\delta \omega_{U}}{\Omega \cos L}+\delta f_{N}+\delta f_{U}\tan L\right)$$

where $k_{g}=g/\bar{g}$ is the gravity acceleration model error scaling factor. The main error source of magnitude LD method is gravity acceleration model error, which is caused by local gravity replaced by the average gravity acceleration. The northward and upward bias of the accelerometer and the upward bias of the gyros also affect the LD error, as well as the location itself.

### 3.2. Geometric Latitude Determination Method

Substituting Equation (21) into Equation (13) yields

(26) $$\begin{array}{c} \sin\left(L+\delta L\right)=\left[{\left(f_{b}+\delta f_{b}\right)}^{T}\left(\omega_{b}+\delta \omega_{b}\right)\right]/\left[\sqrt{{\left(f_{b}+\delta f_{b}\right)}^{T}\left(f_{b}+\delta f_{b}\right)}\sqrt{{\left(\omega_{b}+\delta \omega_{b}\right)}^{T}\left(\omega_{b}+\delta \omega_{b}\right)}\right] \end{array}$$

The following is the derivation and approximation of the first square root reciprocal of Equation (26). In the process of error derivation, only the first-order error terms are retained and the second-order error terms are ignored.

(27) $$\begin{array}{c} \frac{1}{\sqrt{{\left(f_{b}+\delta f_{b}\right)}^{T}\left(f_{b}+\delta f_{b}\right)}}=\frac{1}{\sqrt{f{{}_{b}}^{T}f_{b}+f{{}_{b}}^{T}\delta f_{b}+\delta f{{}_{b}}^{T}f_{b}+\delta f{{}_{b}}^{T}\delta f_{b}}}=\frac{1}{g\sqrt{1+\frac{f{{}_{b}}^{T}\delta f_{b}+\delta f{{}_{b}}^{T}f_{b}+\delta f{{}_{b}}^{T}\delta f_{b}}{g^{2}}}}\\ =\frac{1}{g}{\left(1+\frac{2f{{}_{b}}^{T}\delta f_{b}+\delta f{{}_{b}}^{T}\delta f_{b}}{g^{2}}\right)}^{-\frac{1}{2}}\approx \frac{1}{g}\left(1-\frac{1}{2}\frac{2f{{}_{b}}^{T}\delta f_{b}+\delta f{{}_{b}}^{T}\delta f_{b}}{g^{2}}\right)\approx \frac{1}{g}\left(1-\frac{f{{}_{b}}^{T}\delta f_{b}}{g^{2}}\right) \end{array}$$

Similarly, the derivation and approximation of the second square root reciprocal of Equation (26) can be obtained as follows.

(28) $$\frac{1}{\sqrt{{\left(\omega_{b}+\delta \omega_{b}\right)}^{T}\left(\omega_{b}+\delta \omega_{b}\right)}}\approx \frac{1}{\omega_{b}}\left(1-\frac{\omega {{}_{b}}^{T}\delta \omega_{b}}{\omega {{}_{b}^{2}}_{}}\right)$$

Then the error of geometric LD method becomes

(29) $$\begin{array}{c} \delta L\approx \frac{1}{g_{p}\Omega \cos L}\left({f_{b}}^{T}\delta \omega_{b}+{\delta f_{b}}^{T}\omega_{b}-\frac{{f_{b}}^{T}\omega_{b}{f_{b}}^{T}\delta f_{b}}{{g_{p}}^{2}}-\frac{{f_{b}}^{T}\omega_{b}{\omega_{b}}^{T}\delta \omega_{b}}{\Omega^{2}}\right) \end{array}$$

By substituting Equation (22) into Equation (29), the following geometric LD error equation can be obtained.

(30) $$\delta L=\frac{1}{\Omega }\cos L\delta \omega_{U}-\frac{1}{\Omega }\sin L\delta \omega_{N}\;+\;\frac{1}{g}\delta f_{N}$$

By error analyzing the geometric LD method, according to Equation (30), it can be found that the latitude error is mainly affected by the upward and northward bias of gyros and northward bias of accelerometer, as well as being modulated by the latitude itself.

### 3.3. Analytic 1 Latitude Determination Method

From Equation (15), it is easy to get sine and cosine expressions of pitch θ and roll γ related to IMU output, and substitute these expressions into Equation (18). The LD equation of analytic 1 method can be rewritten as follows.

(31) $$L=\arcsin\frac{\left(\omega_{x}f_{x}+\omega_{y}f_{y}+\omega_{z}f_{z}\right)}{\Omega \sqrt{{\left(f_{x}\right)}^{2}+{\left(f_{y}\right)}^{2}+{\left(f_{z}\right)}^{2}}}=\arcsin\frac{{f_{b}}^{T}\omega_{b}}{\Omega \sqrt{{f_{b}}^{T}f_{b}}}$$

Then the error of latitude can be written as follows

(32) $$\sin\left(L+\delta L\right)=\frac{{\left(f_{b}+\delta f_{b}\right)}^{T}\left(\omega_{b}+\delta \omega_{b}\right)}{\Omega \sqrt{{\left(f_{b}+\delta f_{b}\right)}^{T}\left(f_{b}+\delta f_{b}\right)}}$$

By rearranging Equation (32) in combination with Equations (22) and (27), the latitude error equation of analytic 1 LD method can be expressed as follows

(33) $$\delta L=\frac{1}{\Omega \cos L}\delta \omega_{U}+\frac{1}{g}\delta f_{N}$$

According to Equation (33), both the northward bias of accelerometer and the upward bias of gyros contribute to the latitude error of the analytic 1 LD method.

### 3.4. Analytic 2 Latitude Determination Method

Similar to the derivation process of latitude error in analytic 1 method, it is easy to obtain the sine and cosine expressions between azimuth ψ and IMU output from Equation (19). Associating Equations (15), (19) and (20), the LD equation of analytical 2 method can be rewritten as follows:

(34) $$\begin{array}{c} tanL=f_{b}^{T}\omega_{b}\varsigma \sqrt{\beta^{2}+g_{p}^{2}\alpha^{2}}/\left[\left(-f_{z}\beta g_{p}-f_{x}f_{y}\beta \right)\omega_{x}+\beta \varsigma^{2}\omega_{y}\right.\left.+\left(f_{x}g_{p}^{2}\alpha -f_{y}f_{z}g_{p}\alpha \right)\omega_{z}\right] \end{array}$$

where $\varsigma =\sqrt{{\left(f_{x}^{b}\right)}^{2}+{\left(f_{z}^{b}\right)}^{2}}$, $\alpha =f_{z}^{b}\omega_{ibx}^{b}-f_{x}^{b}\omega_{ibz}^{b}$ and $\beta =-f_{x}^{b}f_{y}^{b}\omega_{ibx}^{b}+\varsigma^{2}\omega_{iby}^{b}-f_{z}^{b}f_{y}^{b}\omega_{ibz}^{b}$.

The derivation of the latitude error is rather complicated. The LD error equation of Analytic 2 are not carried out. As a suggestion for future work, we intend to derive error equations similar to other LD methods. So far, the error analysis is carried out by using the subsequent simulation and experimental results.

## 4. Imu Partial Bias Estimation Algorithm

Inspired by the IMU bias estimation method in [18], the CA-CBE method performed after the latitude determination. The specific calculation process is to calculate the analytical CA matrix ${\hat{C}}_{b}^{n}$ according to Equation (2).

(35) $${\hat{E}}_{s}=\frac{1}{2}\left[{\hat{C}}_{b}^{n}{\left({\hat{C}}_{b}^{n}\right)}^{T}-I\right]$$

where ${\hat{E}}_{s}$ is a symmetric matrix representing the attitude matrix normality error vector $\hat{\eta }$ and orthogonality error vectors $\hat{o}$, respectively, that is

(36) $${\hat{E}}_{s}=\left[\begin{array}{ccc} {\hat{\eta }}_{E} & {\hat{o}}_{U} & {\hat{o}}_{E}\\ {\hat{o}}_{U} & {\hat{\eta }}_{N} & {\hat{o}}_{N}\\ {\hat{o}}_{E} & {\hat{o}}_{E} & {\hat{\eta }}_{U} \end{array}\right]$$

According to the bias estimation Equations (45)–(47) in [18], the upward bias of accelerometer, northward bias and upward bias of the gyros can be computed as follows.

(37) $$\left\{\begin{array}{c} \delta {\hat{f}}_{U}=g_{p}{\hat{\eta }}_{U}\\ \delta {\hat{\omega }}_{N}=\Omega \sin L\left({\hat{\eta }}_{E}-{\hat{\eta }}_{U}\right)\\ \delta {\hat{\omega }}_{U}=\Omega \cos L\left(2{\hat{o}}_{E}-\tan L{\hat{\eta }}_{U}\right) \end{array}\right.$$

From the previous error analysis of the LD method, it can be found that the LD error mainly originates from the IMU bias, and the northward bias of accelerometer, upward and northward biases of gyro are the main error sources. By estimating and compensating for the partial bias of the IMU, the accuracy of the LD can be improved.

## 5. Simulation Results

In order to verify the proposed LD methods and error analysis, corresponding simulation experiments were carried out. The simulation experiments included four aspects as follows.

### 5.1. Multiple Latitude Determination Tests at the Same Location

For the purpose of the test, a navigation grade INS with three gyros and accelerometers was taken as a candidate simulation SINS. The accelerometer data sets were corrupted by 100 μg of constant biases and 10 μg/$\sqrt{h}$ of random walk, and the gyros data sets were added 0.01${}^{{}^{\circ}}$/h of constant biases and 0.001${}^{{}^{\circ}}/\sqrt{h}$ of random walk, respectively [19,20]. The initial position of the simulation SINS was located in Beijing of China with a latitude of 39.97${}^{{}^{\circ}}$ (N), longitude of 116.34${}^{{}^{\circ}}$ (E) and altitude of 50 m. Then, the 5 min data sets sampled with 100 Hz were generated.

**Figure 2:**
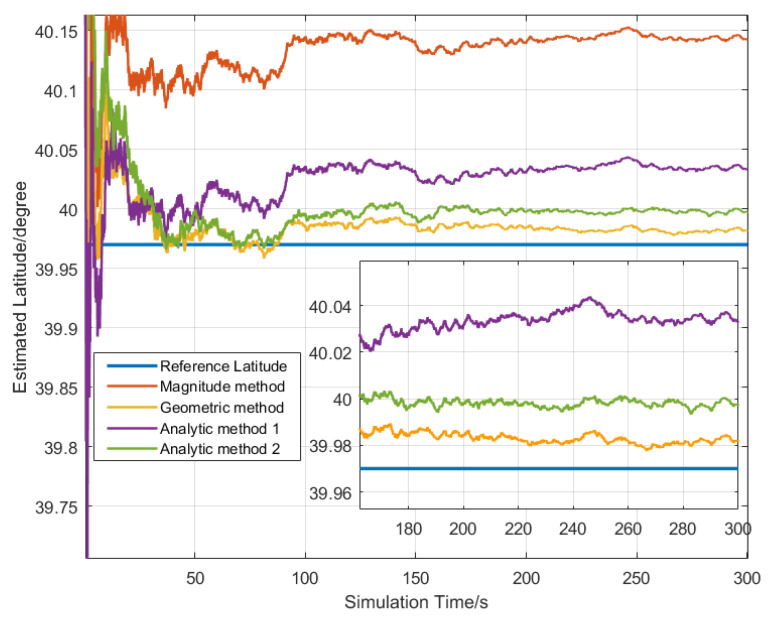
Latitude estimation convergence curve for LD methods.

The 500 Monte Carlo LD simulation tests were performed at the same location. The statistical results are listed in Table 1. The latitude estimated convergence curves by four different LD methods are shown in Figure 2. According to Table 1, it can be seen that the latitude error calculated by the magnitude method at the simulated latitude was 9.91 arcmin, while the latitude error calculated by the other three methods was relatively small (latitude error was less than 3.36 arcmin). The simulated latitude error was very close to the error calculated by the LD error equations. The latitude error obtained by the magnitude method was the largest, and the geometrical method can obtain the smallest LD error in this simulation condition. As shown in Figure 2, the estimated latitude converged at 50 s, and the latitudes estimated by the three LD methods eventually converged to the reference latitude within 5 min. It shows that the LD methods can effectively determine latitude using static IMU output.

### 5.2. The IMU Biases Estimation and Biases Compensation Latitude Determination Test

**Figure 3:**
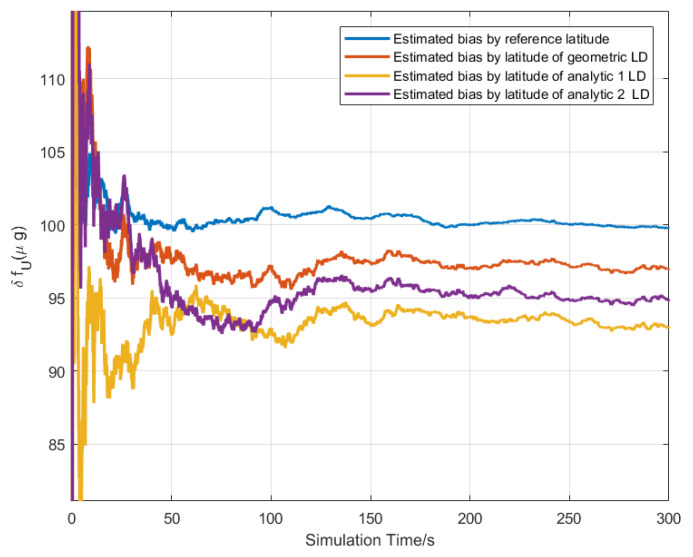
The bias estimation curve of the upward accelerometer by latitude of different LD method.

**Figure 4:**
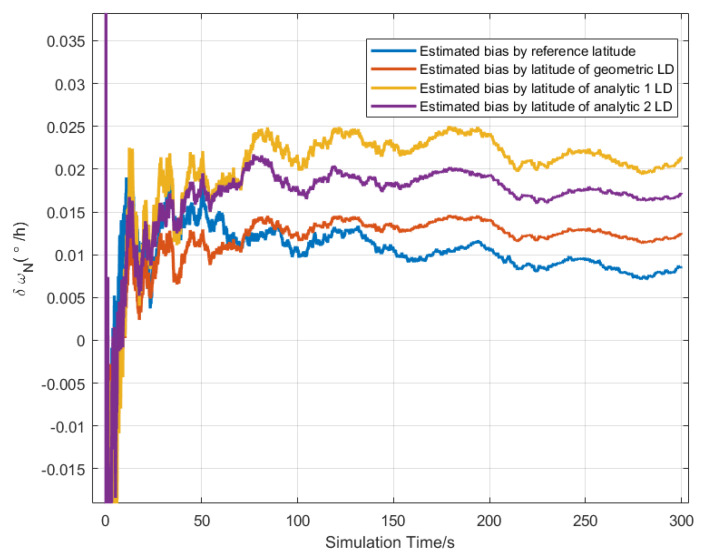
The bias estimation curve of the northward gyro by latitude of different LD method.

**Figure 5:**
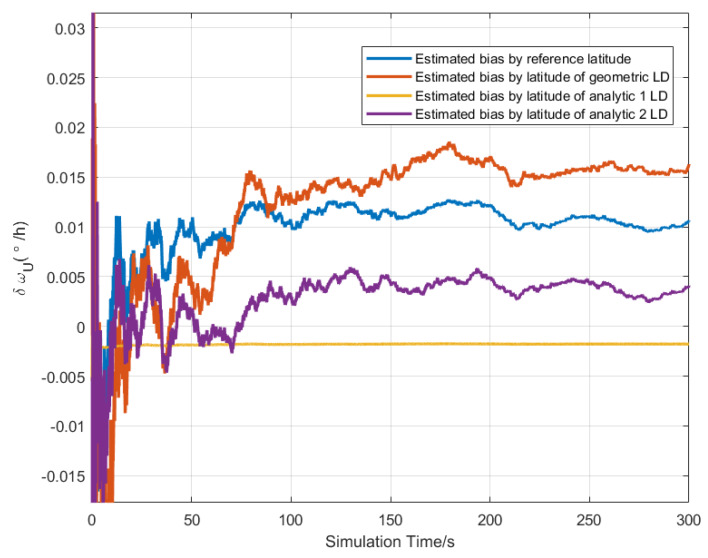
The bias estimation curve of the upward gyro by latitude of different LD method.

The CA-CBE algorithm was used to simultaneously estimate the northward, upward gyros bias, and upward accelerometer bias according to Equation (37), and the simulation conditions were the same as Section 5.1. The bias estimated curve is shown in Figure 3, Figure 4 and Figure 5. The final value of the estimated bias is listed in Table 2. As shown in Table 2, the estimated upward biases of the accelerometer by three LD methods are 96.97 μg, 92.98 μg and 94.84 μg. Similarly, this also can estimate the north and upward gyro bias. However, only the latitude estimated by the geometric LD method can be used to estimate the upward and northward bias of the gyros, as shown in bold values in Table 2. While the latitude calculated by analytic methods cannot be estimated. This also verified that the latitude error estimated by the geometric method was the smallest at the simulated position.

In order to further verify the bias estimation algorithm, a comparison test of LD method with uncompensated and compensated IMU bias was performed. Table 3 shows the latitude errors of uncompensated bias and compensated bias. After the bias was compensated, the latitude error was significantly reduced. Especially for the geometric LD method, it is obvious that after compensating the estimated bias, the LD accuracy was greatly improved.

### 5.3. The Latitude Determination Test at Different Positions

**Figure 6:**
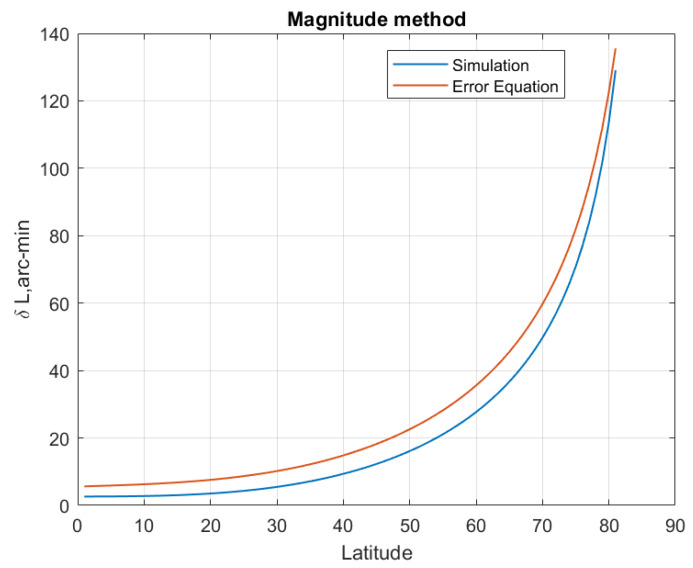
The latitude error of the magnitude method with latitude increasing.

**Figure 7:**
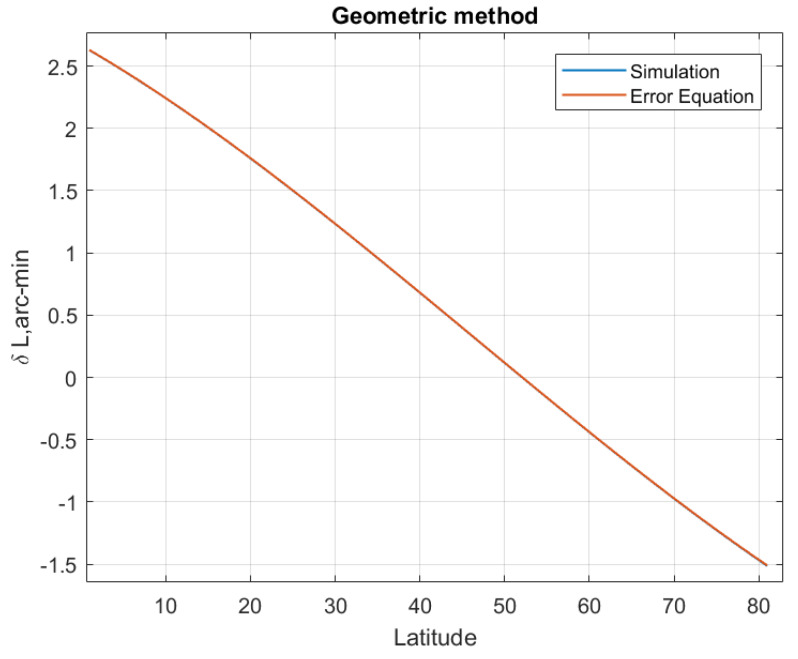
The latitude error of the geometric method with latitude increasing.

**Figure 8:**
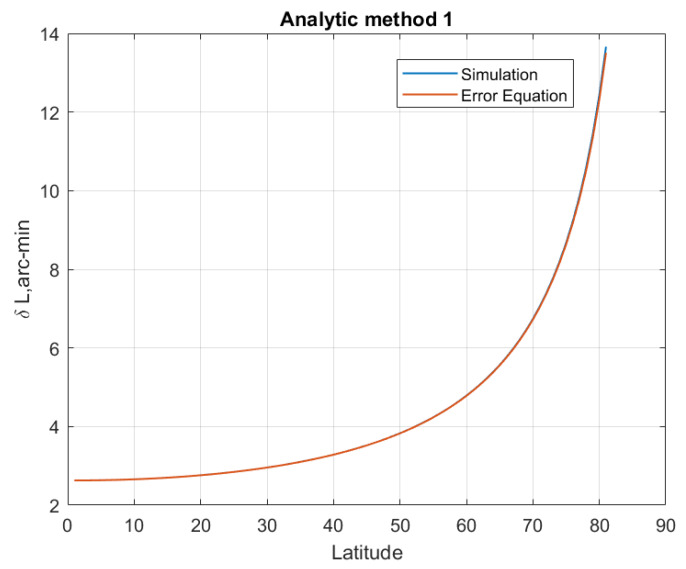
The latitude error of the analytic method 1 with latitude increasing.

**Figure 9:**
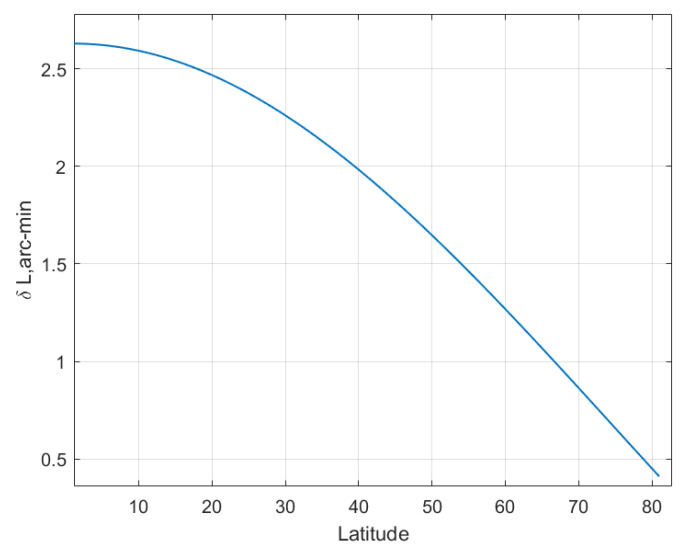
The latitude error of the analytic method 2 with latitude increasing.

The LD error equation indicates that the LD error is affected by the latitude where SINS is located. Suppose SINS is located in the northern hemisphere with a latitude range of 0${}^{{}^{\circ}}$ to 80${}^{{}^{\circ}}$, and the latitude starts from 0${}^{{}^{\circ}}$ with a step size of 1${}^{{}^{\circ}}$. The other simulation conditions are the same as Section 5.1 excepting the location of the SINS. At each simulated latitude, 500 Monte Carlo LD tests are performed, and the average latitude estimation errors are plotted in Figure 6, Figure 7, Figure 8 and Figure 9. The latitude error computed from the Equations (25), (30) and (33) are ploted in Figure 7, Figure 8 and Figure 9. From the Figure 6, Figure 7 and Figure 8, we can conclude that the simulation results are consistent with the error analysis results. Figure 9 only shows the latitude error results of analytical method 2, which provides a numerical reference for us to determine the latitude. The latitude error of the magnitude method and the analytical 1 method is mainly affected by $\epsilon_{U}/\Omega \cos L$, so the estimated latitude error will increase rapidly as the location of the SINS increases. However, the magnitude method is affected by $g_{p}\nabla_{U}\tan L/\bar{g}$, so its latitude error increases much more than the others method. The latitude estimation error of the geometric method is between −2 arcmin and 2 arcmin. The latitude error of geometric method decreases with increasing latitude, and the estimated latitude error is zero at a certain latitude point. Analytical method 2 is less affected by latitude than the others method, its latitude error is smaller than other methods when SINS is in a high latitude area. The simulation results in the southern hemisphere have symmetrical transparent feature.

### 5.4. The Latitude Determination Test by Different Level of IMU

To verify the applicability of the LD methods, the simulation experiments on IMUs of different accuracy levels were conducted. The constant bias of the gyro was 1${}^{{}^{\circ}}$/h, 0.5${}^{{}^{\circ}}$/h, 0.02${}^{{}^{\circ}}$/h, 0.001${}^{{}^{\circ}}$/h, respectively, and the corresponding constant bias of the accelerometer was 1000 μg, 500 μg, 100 μg, 10 μg, respectively. The simulation location was the same as Section 5.1, 500 Monte Carlo LD tests were performed, and the average latitude estimation errors are listed in Table 4. Obviously, error of LD will shapely decline with performance of IMU incline, the simulation LD error is also close to the error computed by the LD error equations.

## 6. Experimental Results

To further verify the LD methods in practical applications, the experiments was carried out on an SINS manufactured in our laboratory. The SINS was developed using three dithered ring laser gyroscopes and three quartz accelerometers, and the detail specifications of the SINS are shown in Table 5. The experiments mainly included two aspects, one was multiple LD tests at the same location and the other was LD test at multiple locations.

### 6.1. Multiple Latitude Determination Experiments at the Same Position

**Figure 10:**
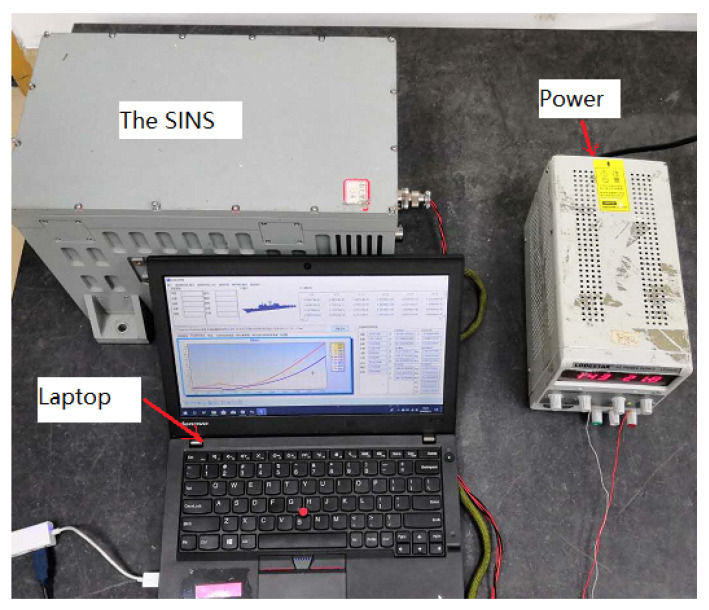
Experimental equipment.

The multiple LD testes were implemented in the (Inertial Technology and Integrated Navigation Laboratory) New Main Building of BeiHang University in Haidian District Beijing, China. The experiment equipment mainly included an SINS, a 24 V DC power supply, a marble platform and a laptop for raw data sampling at 200 Hz, as shown in Figure 10. The geographic latitude of the site were 39.977200${}^{{}^{\circ}}$ (N).

The system needed to be warmed up for 30 min before the start of the test. Then the LD test was implemented by collecting 5 min static raw data multiple times.

**Figure 11:**
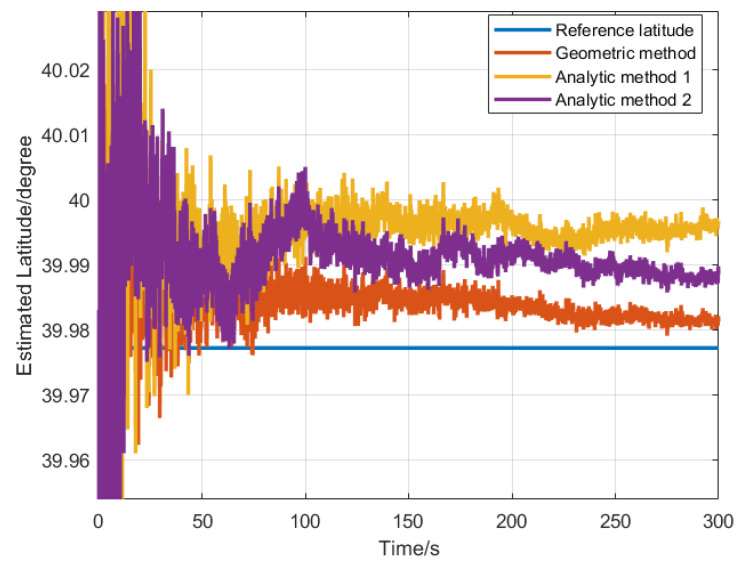
The latitude estimation curve of real INS test.

According to the LD algorithm and its error analysis, the geometric method, the analytical method 1 and the analytical method 2 were chosen to compute latitude. The estimated geographic latitude of 7 times is shown in Table 6 and one of the curves of three LD methods with time is shown in Figure 11, respectively. In the process of calculating latitude, the CA-CBE algorithm was used to estimate the bias of northward gyro, the bias of upward gyro and the upward bias of the accelerometer, the estimated biases are shown in Table 7.

Then, the LD process was performed again after compensating the estimated biases. The statistical latitude estimation errors for uncompensated and compensated biases are listed in Table 8. As is evident from Table 8, the average latitude errors of the proposed methods were −0.83 arcmin, −1.23 arcmin and −1.02 arcmin, which is consistent with the previous error analysis results. After compensating the biases, the estimated latitude accuracy was significantly improved, which also proves the effectiveness of the bias estimation method. The mean values of latitude errors after compensating the biases were reduced to 0.03 arcmin, 0.21 arcmin and 0.29 arcmin, falling by 96.37%, 82.88% and 71.48% correspond to the geometric method, the analytical 1 method and the analytical 2, respectively.

### 6.2. Latitude Determination Experiments at the Multiple Positions

**Figure 12:**
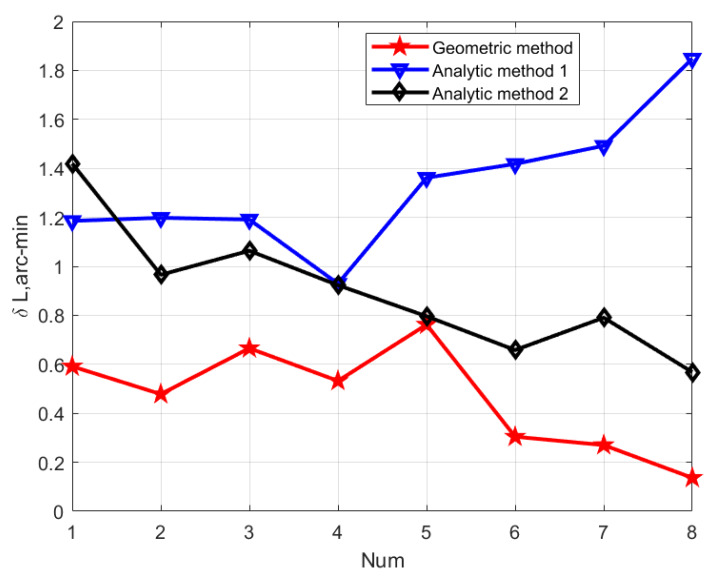
The latitude error curve of real INS for diferent location in China.

To further validate the capability, the same RLG SINS is applied in LD experiments at the multiple positions. We chose eight different positions in China for LD experiments. The latitudes of the multiple positions ranged from 26.0${}^{{}^{\circ}}$ to 40.7${}^{{}^{\circ}}$. These eight positions were located in Fuzhou City, Xi’an City (2 points), Lianyungang City, Zhengzhou City, Beijing City (2 points) and Huludao City, respectively. The estimated geographic latitudes of the eight different positions are shown in Table 9. Figure 12 illustrates the latitude error computed for the three LD methods, and the x-axis presents the eight positions increasing with latitude meanwhile the *y*-axis is the latitude error. It is indicated from Figure 12 that the variation of latitude estimation error with increased latitude occurred in the same manner as in Figure 7, Figure 8 and Figure 9. This is consistent with the latitude error equation. It is consistent with the previous LD error equation.

## 7. Conclusions

This paper mainly solves the problem that the INS cannot be started in some unknown positions (especially the latitude). This technology can be used to determine the latitude information of the INS, and the LD is performed only based on the output of the static inertial navigation system itself. Four algorithms of LD are comprehensively introduced, and the relationship between the LD error, IMU bias and INS position is emphatically deduced. Simulation and experiments verify the effectiveness of the LD algorithm, as well as the derived error equations. For gyros and accelerometers with bias of 0.01${}^{{}^{\circ}}$/h and 100 μg, the LD error of 500 monte carlo simulation of geometric method is less than 1 arc-min, while for gyros and accelerometers with bias of 1${}^{{}^{\circ}}$/h and 1 mg, the LD error is less than 0.5${}^{{}^{\circ}}$, indicating that the feasibility of extending the method to lower grade IMU. The “CA-CBE” bias estimation algorithm is introduced into the LD stage, which can effectively estimate the partial bias of IMU. After the estimated IMU bias is compensated, the accuracy of the LD is further improved. The LD error of actual SINS is less than 1.5 arc-min when the bias of IMU is uncompensated, and the LD error is less than 0.3 arc-min after compensated bias of IMU. Finally, the LD error is affected by the position of the inertial navigation system. The typical navigation-level SINS is used for imulation and experimental verification the different latitudes. The results are consistent with the derived error formula. The application of this technology can further improve the autonomy of INS.

## Figures and Tables

**Table 1:** The 500 Monte Carlo latitude determination (LD) simulation results of latitude error for navigation-grade inertial navigation system (INS) (the reference latitude is 39.97${}^{{}^{\circ}}$).

Method	Max Value	Min Value	Mean Value	Simulation Error	Error Equation	STD
Magnitude	40.19175920	40.08608037	40.13512112	9.91	15.46	1.075
Geometric	40.01979780	39.93571042	39.98050184	0.63	0.626	0.815
Analytic 1	40.08254491	39.97725655	40.02612170	3.36	3.326	1.072
Analytic 2	40.04296086	39.95299871	40.00248764	1.94	—	0.908

**Table 2:** The biases estimation by different LD methods.

Method	$\nabla_{N}$ (μg)	$\varepsilon_{N}$ (${}^{{}^{\circ}}/h$)	$\varepsilon_{U}$ (${}^{{}^{\circ}}/h$)
Reference	99.80	0.0091	0.01065
Geometric	96.97	**0.0124**	**0.0156**
Analytic 1	92.98	0.0214	−0.0018
Analytic 2	94.84	0.0173	0.00408

**Table 3:** The latitude error of biases uncompensated and compensated case (the unit is arcmin).

	Magnitude	Geometric	Analytic 1	Analytic 2
uncompensated	10.32	0.72	3.78	1.66
compensated	6.81	0.094	0.57	1.03

**Table 4:** The latitude error of different level biases of the IMU.

LD Error(arc-min)	Simulation Error				Equation Error		
Biases of IMU	Magnitude	Geometric	Analytic 1	Analytic 2	Maginitude	Geometric	Analytic 1
(1${}^{{}^{\circ}}$/h, 1000 μg)	333.83	27.38	322.78	180.67	367.10	31.78	301.67
(0.5${}^{{}^{\circ}}$/h, 500 μg)	158.66	13.15	150.26	87.67	186.69	15.89	150.83
(0.02${}^{{}^{\circ}}$/h, 100 μg)	12.74	0.80	6.19	3.65	18.45	0.91	6.31
(0.001${}^{{}^{\circ}}$/h, 10 μg)	6.60	0.07	0.33	0.20	7.20	0.06	0.33

**Table 5:** Specifications of the RLG IMU.

Characteristics	RLG IMU
Gyros constant bias	0.005${}^{{}^{\circ}}$/h
Gyros random walk	0.0005${}^{{}^{\circ}}$/h${}^{1/2}$
Accelerometers constant bias	20 μg
Accelerometers random walk	10 μg/h${}^{1/2}$

**Table 6:** The LD results at the same position. (The reference geographic latitude is 39.977200${}^{{}^{\circ}}$ (N)).

No.	Geometric	Analytic 1	Analytic 2
1	39.97759574	39.98072908	39.98273388
2	39.97750989	39.98061890	39.98093766
3	39.97727617	39.98069921	39.98128569
4	39.97771365	39.98071466	39.98267036
5	39.97724045	39.98076147	39.98078857
6	39.97773299	39.98076867	39.98234140
7	39.97843692	39.98084315	39.98379346
**Mean vaule**	39.97764369	39.98073359	39.98207872

**Table 7:** The estimated biases of SINS.

No.	1	2	3	4
$\varepsilon_{N}$ (${}^{{}^{\circ}}$/h)	0.0038	0.0026	0.0036	0.0033
$\varepsilon_{U}$ (${}^{{}^{\circ}}$/h)	−0.0048	−0.0050	−0.0046	−0.0052
$\nabla_{N}$(μg)	35.48	35.46	35.88	35.82
**No.**	**5**	**6**	**7**	**Mean value**
$\varepsilon_{N}$ (${}^{{}^{\circ}}$/h)	0.0044	0.0033	0.0019	0.0033
$\varepsilon_{U}$ (${}^{{}^{\circ}}$/h)	−0.0038	−0.0048	−0.0056	−0.0048
$\nabla_{N}$(μg)	36.30	36.37	37.14	36.06

**Table 8:** The latitude errors for uncompensated and compensated biases (The unit is arcmin).

No.	Uncompensated			Compensated		
	**Geometric**	**Analytic 1**	**Analytic 2**	**Geometric**	**Analytic 1**	**Analytic 2**
1	−1.07	−1.21	−1.11	0.024	0.212	0.332
3	−0.61	−1.28	−0.92	0.019	0.205	0.224
3	−0.71	−1.16	−1.04	0.005	0.210	0.245
4	−0.76	−1.34	−1.06	0.031	0.211	0.328
5	−0.70	−0.92	−1.09	0.002	0.214	0.215
6	−1.06	−1.23	−1.03	0.032	0.214	0.308
7	−0.88	−1.45	−0.86	0.074	0.219	0.396
**Mean**	−0.83	−1.23	−1.02	0.03	0.21	0.29

**Table 9:** The LD results of diferent positions. (The unit is degree).

No.	Geometric	Analytic 1	Analytic 2	Reference
1	26.04986553	26.05976811	26.06367110	26.04000920
2	34.01732031	34.02931969	34.02544198	34.00934964
3	34.21044224	34.21919980	34.21707603	34.19934832
4	34.65423605	34.66085117	34.66074875	34.64536500
5	34.81864047	34.82863485	34.81920788	34.80593966
6	39.98227452	40.00083623	39.98817058	39.97720000
7	40.12938190	40.14974919	40.13805272	40.12487900
8	40.70085247	40.72938171	40.70804533	40.69857790

## References

[1] Savage P.G. (2007). Strapdown Aanalytics.

[2] Titterton D.H., Weston J.L. (2004). Strapdown Inertial Navigation Technology.

[3] Silva F.O., Hemerly E.M., Filho W.C.L. (2016). Error Analysis of analytical coarse alignment formulations for stationary SINS. IEEE Trans. Aerosp. Electron. Syst..

[4] Silva F.O., Hemerly E.M., Filho W.C.L. Influence of Latitude in Coarse Self-Alignment of Strapdown Inertial Navigation Systems. Proceedings of the 2014 IEEE/ION Position, Location and Navigation Symposium.

[5] Qin Y.Y. (2006). Inertial Navigation.

[6] Britting K.R. (1997). Inertial Navigation System Analysis.

[7] Silva F.O., Filho W.C.L., Hemerly E.M. (2015). Design of a Stationary Self-Alignment Algorithm for Strapdown Inertial Navigation Systems. Ifac Pap..

[8] Jiang Y. (1998). Error analysis of analytic coarse alignment methods. IEEE Trans. Aerosp. Electron. Syst..

[9] Wang X., Shen G. (2005). A fast and accurate initial alignment method for strapdown inertial navigation system on stationary base. J. Control. Theory Appl..

[10] Yang H.T., Zhou B., Wang L.X., Wei Q., Zhang R. (2019). A Novel Method for Fast Stationary Initial Alignment Based on Extended Measurement Information. IEEE Access.

[11] Wu M., Wu Y., Hu X., Hu D. (2011). Optimization-based alignment for inertial navigation systems: Theory and algorithm. Aerosp. Sci. Technol..

[12] Yan G., Xu D., Jiang H. (2008). SINS Initial Alignment Analysis Under Geographic Latitude Uncertainty. Aerosp. Control.

[13] Zheng Z.Y., Zhou A.J., Tang J., Xu X.B. (2019). An Initial Alignment Method of SINS without Latitude Based on Calculation of Earth Axis Vector. J. Astronaut..

[14] Zheng Z.Y., Xie W.H., Zheng Z.L. (2019). An New Alignment Method of SINS Without Latitude Based on Quaternion Calculation. Aerosp. Control.

[15] Wang Y., Yang J., Yang B. (2012). SINS Initial Alignment of Swaying Base Under Geographic Latitude Uncertainty. Acta Aeronaut. Astronaut. Sin..

[16] Lv W., Chen X. (2017). Novel self-alignment algorithm with unknown latitude for SINS on swing base. J. Chin. Inert. Technol..

[17] Li J., Fang J., Du M. (2012). Error Analysis and gyro-bias calibration of analytic coarse alignment for airborne POS. IEEE Trans. Instrum. Meas..

[18] Silva F.O., Hemerly E.M., Filho W.C.L., Kuga H.K. (2018). A Fast In-Field Coarse Alignment and Bias Estimation Method for Stationary Intermediate-Grade IMUs. IEEE Trans. Instrum. Meas..

[19] Wang S.E., Yang G.L., Wang L.F. (2019). An Improve Hybrid Calibration Scheme for Strapdown Inertial Navigation System. IEEE Access.

[20] Acharya A., Sadhu S., Ghoshal T.K. (2011). Improved self-alignment scheme for SINS using augmented measurement. Aerosp. Sci. Technol..

